# Hexa­kis­(dimethyl sulfoxide-κ*O*)nickel(II) bis­(2,2-dicyano­ethene-1,1-dithiol­ato-κ^2^ *S*,*S*′)nickelate(II)

**DOI:** 10.1107/S1600536811053827

**Published:** 2011-12-21

**Authors:** Meiju Niu, Shumei Fan, Guihua Liu

**Affiliations:** a School of Chemistry and Chemical Engineering, Liaocheng University, Shandong 252059, People’s Republic of China; b Dongchang College Liaocheng University, Shandong 252000, People’s Republic of China

## Abstract

The reaction of NiCl_2_·6H_2_O with sodium 2,2-dicyano­ethene-1,1-dithiol­ate [Na_2_(*i*-mnt)] in dimethyl sulfoxide produces the title complex, [Ni(C_2_H_6_OS)_6_][Ni(C_4_N_2_S_2_)_2_]. There is half each of an [Ni(C_2_H_6_OS)_6_]^2−^ complex anion and an [Ni{(CH_3_)_2_SO}_6_]^2+^ complex cation in the asymmetric unit. The *i*-mnt ligand coordinates in a bidentate manner to the Ni atom in the anion through the two chelating S atoms in an approximate square-planar geometry. The Ni atom in the complex cation has an octahedral coordination environment with six dimethyl sulfoxide mol­ecules as ligands.

## Related literature

For related structures, see Gao *et al.* (2004 ▶, 2005 ▶); Yu *et al.* (2005 ▶); Chen & Yu (2005 ▶).
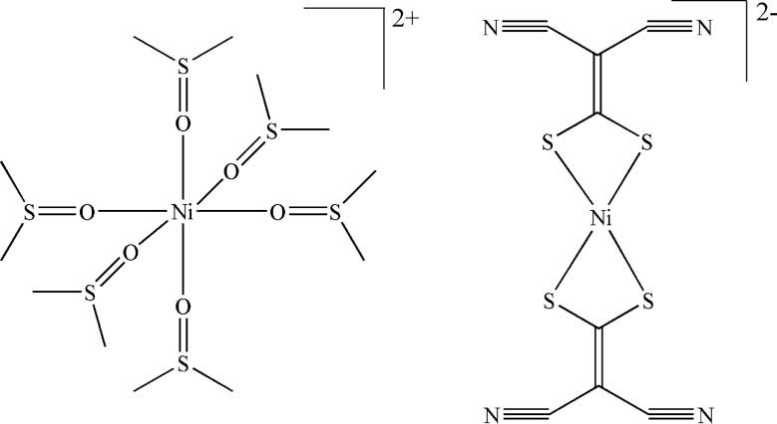


## Experimental

### 

#### Crystal data

[Ni(C_2_H_6_OS)_6_][Ni(C_4_N_2_S_2_)_2_]
*M* *_r_* = 866.55
Monoclinic, 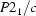

*a* = 8.3368 (10) Å
*b* = 12.6763 (17) Å
*c* = 18.710 (2) Å
β = 102.466 (2)°
*V* = 1930.6 (4) Å^3^
*Z* = 2
Mo *K*α radiation
μ = 1.55 mm^−1^
*T* = 298 K
0.50 × 0.48 × 0.05 mm

#### Data collection

Bruker SMART CCD area-detector diffractometer
Absorption correction: multi-scan (*SADABS*; Sheldrick, 1996 ▶) *T*_min_ = 0.511, *T*_max_ = 0.921
9458 measured reflections
3379 independent reflections
2328 reflections with *I* > 2σ(*I*)
*R*_int_ = 0.054

#### Refinement

*R*[*F* ^2^ > 2σ(*F* ^2^)] = 0.038
*wR*(*F* ^2^) = 0.095
*S* = 1.06
3379 reflections
199 parameters
H-atom parameters constrained
Δρ_max_ = 0.30 e Å^−3^
Δρ_min_ = −0.35 e Å^−3^

### 

Data collection: *SMART* (Bruker, 2007 ▶); cell refinement: *SAINT* (Bruker, 2007 ▶); data reduction: *SAINT*; program(s) used to solve structure: *SHELXS97* (Sheldrick, 2008 ▶); program(s) used to refine structure: *SHELXL97* (Sheldrick, 2008 ▶); molecular graphics: *SHELXTL* (Sheldrick, 2008 ▶); software used to prepare material for publication: *SHELXTL*.

## Supplementary Material

Crystal structure: contains datablock(s) I, global. DOI: 10.1107/S1600536811053827/vm2141sup1.cif

Structure factors: contains datablock(s) I. DOI: 10.1107/S1600536811053827/vm2141Isup2.hkl

Additional supplementary materials:  crystallographic information; 3D view; checkCIF report

## Figures and Tables

**Table:** 

Ni1—O3	2.052 (2)
Ni1—O2	2.060 (3)
Ni1—O1	2.064 (2)
Ni2—S4	2.2010 (11)
Ni2—S5	2.2030 (11)

**Table:** 

O3—Ni1—O2	90.14 (11)
S4—Ni2—S5	78.96 (4)

**Fig. 1.**
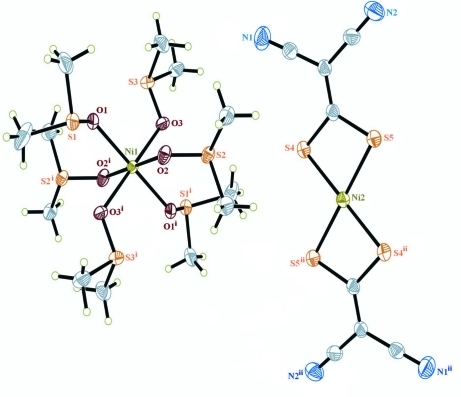
The molecular structure of the title compound with atom labels and 40% probability displacement ellipsoids for non-H atoms. Symmetry codes: (i) -x + 1,-y + 1,-z + 1, (ii) -x + 2, -y, -z + 1.

## supplementary crystallographic information

### Comment

The bridging ligand 1,1-dicyanoethene-2,2-dithiolate has been attracting more and more attention due to its delocalized

π electron system able to build special planar conjugated structures (Yu *et al.*, 2005). The title complex consists of one [Ni(*i*-mnt)_2_]^2-^ (where *i*-mnt is 1,1-dicyanoethene-2,2-dithiolate) complex anion and one [Ni((CH_3_)_2_SO)_6_]^2+^ complex cation. For the [Ni(*i*-mnt)_2_]^2-^ complex anion, the NiS_4_ group is square planar and tortiled slightly at an angle of 2.90 (14)° with respect to the plane of *i*-mnt ligand (Chen *et al.*, 2005). FourNi-S bonds present two comparable distances of 2.2010 (11) and 2.2030 (11)Å, similar to the Ni-S distance of 2.172 (3)

Å in [K(N18C6)]_2_[Ni(mnt)_2_] (Gao *et al.*, 2004). The average S-C, C≡N, C-C and C=C bond lengths were 1.717, 1.141, 1.418, 1.379 Å, respectively (Gao *et al.*, 2005). The [Ni((CH_3_)_2_SO)_6_]^2+^ complex cation contains a nickel with six-coordinated octahedral geometry.

### Experimental

The title compound, [Ni((CH_3_)_2_SO)_6_][Ni(*i*-mnt)_2_] was synthesizd by the reaction of 0.05 mmol NiCl_2_.6H_2_O and 0.1 mmol Na_2_(*i*-mnt)(1,1-dicyanoethene-2,2-dithiolate sodium) in 5 ml water. The solution was stirred for 2 hours and then filtered. The precipitate was dissolved in 10ml Dimethyl Sulfoxide. After slow evaporation of the solution over one month, deep green crystals suitable for X-ray diffraction were obtained (56.8%, m.p. 527-529 K).

### Refinement

All H atoms were placed in geometrically idealized positions and treated as riding on their parent atoms, with C—H 0.96 Å, with *U*_iso_(H) =1.2*U*_eq_(C), and refined as riding on their parent atoms.

### Crystal data

**Table:**

C_12_H_36_NiO_6_S_6_^2+^·C_8_N_4_NiS_4_^2^^−^	*F*(000) = 896
*M**_r_* = 866.55	*D*_x_ = 1.491 Mg m^−^^3^
Monoclinic, *P*2_1_/*c*	Mo *K*α radiation, λ = 0.71073 Å
Hall symbol: -P 2ybc	Cell parameters from 2760 reflections
*a* = 8.3368 (10) Å	θ = 2.5–24.3°
*b* = 12.6763 (17) Å	µ = 1.55 mm^−^^1^
*c* = 18.710 (2) Å	*T* = 298 K
β = 102.466 (2)°	Needle, green
*V* = 1930.6 (4) Å^3^	0.50 × 0.48 × 0.05 mm
*Z* = 2	

### Data collection

**Table:**

Bruker SMART CCD area-detector diffractometer	3379 independent reflections
Radiation source: fine-focus sealed tube	2328 reflections with *I* > 2σ(*I*)
graphite	*R*_int_ = 0.054
phi and ω scans	θ_max_ = 25.0°, θ_min_ = 2.0°
Absorption correction: multi-scan (*SADABS*; Sheldrick, 1996)	*h* = −9→9
*T*_min_ = 0.511, *T*_max_ = 0.921	*k* = −12→15
9458 measured reflections	*l* = −22→18

### Refinement

**Table:**

Refinement on *F*^2^	Primary atom site location: structure-invariant direct methods
Least-squares matrix: full	Secondary atom site location: difference Fourier map
*R*[*F*^2^ > 2σ(*F*^2^)] = 0.038	Hydrogen site location: inferred from neighbouring sites
*wR*(*F*^2^) = 0.095	H-atom parameters constrained
*S* = 1.06	*w* = 1/[σ^2^(*F*_o_^2^) + (0.0275*P*)^2^ + 1.0073*P*] where *P* = (*F*_o_^2^ + 2*F*_c_^2^)/3
3379 reflections	(Δ/σ)_max_ = 0.001
199 parameters	Δρ_max_ = 0.30 e Å^−^^3^
0 restraints	Δρ_min_ = −0.35 e Å^−^^3^

### Fractional atomic coordinates and isotropic or equivalent isotropic displacement parameters (Å^2^)

**Table:**

	*x*	*y*	*z*	*U*_iso_*/*U*_eq_	
Ni1	0.5000	0.5000	0.5000	0.02973 (18)	
Ni2	1.0000	0.0000	0.5000	0.0415 (2)	
N1	0.7064 (6)	0.2565 (4)	0.2505 (2)	0.0863 (15)	
N2	1.1494 (6)	0.0945 (4)	0.2174 (2)	0.0751 (13)	
O1	0.4945 (3)	0.64165 (18)	0.44513 (13)	0.0367 (6)	
O2	0.7372 (3)	0.47506 (19)	0.48840 (15)	0.0440 (7)	
O3	0.4122 (3)	0.41769 (19)	0.40520 (13)	0.0436 (7)	
S1	0.62727 (13)	0.72370 (8)	0.46849 (5)	0.0412 (3)	
S2	0.78272 (12)	0.36249 (8)	0.47193 (5)	0.0385 (3)	
S3	0.32535 (13)	0.47016 (8)	0.33476 (5)	0.0391 (3)	
S4	0.81754 (14)	0.08901 (9)	0.41999 (6)	0.0489 (3)	
S5	1.10676 (15)	−0.00988 (9)	0.40228 (6)	0.0533 (3)	
C1	0.5310 (7)	0.8291 (4)	0.5044 (3)	0.095 (2)	
H1A	0.4416	0.8553	0.4675	0.142*	
H1B	0.6092	0.8846	0.5198	0.142*	
H1C	0.4898	0.8047	0.5456	0.142*	
C2	0.6505 (7)	0.7821 (4)	0.3864 (2)	0.0732 (16)	
H2A	0.6983	0.7320	0.3586	0.110*	
H2B	0.7208	0.8426	0.3971	0.110*	
H2C	0.5449	0.8036	0.3586	0.110*	
C3	0.9350 (5)	0.3786 (3)	0.4198 (2)	0.0503 (11)	
H3A	0.8874	0.4120	0.3741	0.075*	
H3B	0.9777	0.3108	0.4106	0.075*	
H3C	1.0224	0.4216	0.4465	0.075*	
C4	0.9093 (6)	0.3174 (4)	0.5552 (2)	0.0585 (13)	
H4A	0.9998	0.3651	0.5701	0.088*	
H4B	0.9504	0.2484	0.5480	0.088*	
H4C	0.8462	0.3144	0.5924	0.088*	
C5	0.2206 (6)	0.3635 (4)	0.2835 (2)	0.0676 (14)	
H5A	0.2961	0.3066	0.2827	0.101*	
H5B	0.1756	0.3863	0.2343	0.101*	
H5C	0.1334	0.3399	0.3057	0.101*	
C6	0.4776 (6)	0.4904 (4)	0.2835 (2)	0.0600 (13)	
H6A	0.5526	0.5440	0.3065	0.090*	
H6B	0.4265	0.5123	0.2348	0.090*	
H6C	0.5365	0.4258	0.2813	0.090*	
C7	0.9525 (5)	0.0750 (3)	0.3630 (2)	0.0409 (10)	
C8	0.9395 (5)	0.1255 (3)	0.2967 (2)	0.0427 (10)	
C9	0.8105 (6)	0.1979 (4)	0.2706 (2)	0.0526 (12)	
C10	1.0561 (6)	0.1086 (3)	0.2528 (2)	0.0522 (12)	

### Atomic displacement parameters (Å^2^)

**Table:**

	*U*^11^	*U*^22^	*U*^33^	*U*^12^	*U*^13^	*U*^23^
Ni1	0.0336 (4)	0.0269 (4)	0.0290 (4)	0.0013 (3)	0.0074 (3)	−0.0017 (3)
Ni2	0.0476 (5)	0.0370 (4)	0.0408 (4)	0.0016 (4)	0.0116 (3)	−0.0019 (3)
N1	0.092 (4)	0.077 (3)	0.076 (3)	0.030 (3)	−0.011 (3)	−0.008 (2)
N2	0.080 (3)	0.093 (3)	0.058 (3)	0.006 (3)	0.029 (2)	0.002 (2)
O1	0.0380 (16)	0.0297 (14)	0.0415 (14)	−0.0024 (12)	0.0067 (12)	0.0023 (12)
O2	0.0373 (17)	0.0336 (16)	0.0633 (18)	−0.0007 (12)	0.0155 (14)	−0.0079 (13)
O3	0.061 (2)	0.0358 (15)	0.0309 (14)	−0.0010 (14)	0.0039 (13)	−0.0047 (12)
S1	0.0402 (7)	0.0377 (6)	0.0461 (6)	−0.0039 (5)	0.0104 (5)	0.0007 (5)
S2	0.0339 (6)	0.0327 (6)	0.0497 (6)	0.0002 (4)	0.0104 (5)	−0.0077 (5)
S3	0.0409 (6)	0.0434 (6)	0.0325 (5)	0.0031 (5)	0.0071 (4)	−0.0050 (4)
S4	0.0502 (8)	0.0503 (7)	0.0486 (6)	0.0102 (5)	0.0157 (5)	−0.0005 (5)
S5	0.0537 (8)	0.0614 (8)	0.0473 (6)	0.0163 (6)	0.0161 (5)	0.0049 (6)
C1	0.094 (5)	0.053 (3)	0.155 (6)	−0.023 (3)	0.067 (4)	−0.048 (4)
C2	0.086 (4)	0.072 (3)	0.063 (3)	−0.032 (3)	0.018 (3)	0.015 (3)
C3	0.045 (3)	0.048 (3)	0.063 (3)	−0.003 (2)	0.022 (2)	−0.011 (2)
C4	0.067 (3)	0.049 (3)	0.056 (3)	0.014 (2)	0.004 (2)	0.000 (2)
C5	0.077 (4)	0.074 (3)	0.049 (3)	−0.025 (3)	0.008 (2)	−0.017 (3)
C6	0.062 (3)	0.064 (3)	0.061 (3)	−0.005 (3)	0.030 (3)	0.006 (2)
C7	0.045 (3)	0.035 (2)	0.041 (2)	−0.0014 (19)	0.0061 (19)	−0.0096 (18)
C8	0.045 (3)	0.042 (3)	0.041 (2)	0.002 (2)	0.008 (2)	−0.0053 (19)
C9	0.066 (3)	0.049 (3)	0.040 (2)	−0.003 (3)	0.005 (2)	−0.010 (2)
C10	0.065 (4)	0.051 (3)	0.040 (3)	−0.002 (2)	0.008 (2)	0.001 (2)

### Geometric parameters (Å, °)

**Table:**

Ni1—O3	2.052 (2)	S5—C7	1.716 (4)
Ni1—O3^i^	2.052 (2)	C1—H1A	0.9600
Ni1—O2^i^	2.060 (3)	C1—H1B	0.9600
Ni1—O2	2.060 (3)	C1—H1C	0.9600
Ni1—O1	2.064 (2)	C2—H2A	0.9600
Ni1—O1^i^	2.064 (2)	C2—H2B	0.9600
Ni2—S4	2.2010 (11)	C2—H2C	0.9600
Ni2—S4^ii^	2.2010 (11)	C3—H3A	0.9600
Ni2—S5^ii^	2.2030 (11)	C3—H3B	0.9600
Ni2—S5	2.2030 (11)	C3—H3C	0.9600
N1—C9	1.142 (6)	C4—H4A	0.9600
N2—C10	1.139 (5)	C4—H4B	0.9600
O1—S1	1.513 (3)	C4—H4C	0.9600
O2—S2	1.525 (3)	C5—H5A	0.9600
O3—S3	1.514 (3)	C5—H5B	0.9600
S1—C2	1.752 (4)	C5—H5C	0.9600
S1—C1	1.765 (5)	C6—H6A	0.9600
S2—C3	1.772 (4)	C6—H6B	0.9600
S2—C4	1.777 (4)	C6—H6C	0.9600
S3—C6	1.767 (4)	C7—C8	1.378 (5)
S3—C5	1.775 (4)	C8—C9	1.418 (6)
S4—C7	1.719 (4)	C8—C10	1.418 (6)
			
O3—Ni1—O3^i^	180.000 (1)	H1B—C1—H1C	109.5
O3—Ni1—O2^i^	89.86 (11)	S1—C2—H2A	109.5
O3^i^—Ni1—O2^i^	90.14 (11)	S1—C2—H2B	109.5
O3—Ni1—O2	90.14 (11)	H2A—C2—H2B	109.5
O3^i^—Ni1—O2	89.86 (11)	S1—C2—H2C	109.5
O2^i^—Ni1—O2	180.00 (15)	H2A—C2—H2C	109.5
O3—Ni1—O1	92.69 (9)	H2B—C2—H2C	109.5
O3^i^—Ni1—O1	87.31 (9)	S2—C3—H3A	109.5
O2^i^—Ni1—O1	90.01 (10)	S2—C3—H3B	109.5
O2—Ni1—O1	89.99 (10)	H3A—C3—H3B	109.5
O3—Ni1—O1^i^	87.31 (9)	S2—C3—H3C	109.5
O3^i^—Ni1—O1^i^	92.69 (9)	H3A—C3—H3C	109.5
O2^i^—Ni1—O1^i^	89.99 (10)	H3B—C3—H3C	109.5
O2—Ni1—O1^i^	90.01 (10)	S2—C4—H4A	109.5
O1—Ni1—O1^i^	180.0	S2—C4—H4B	109.5
S4—Ni2—S4^ii^	180.00 (5)	H4A—C4—H4B	109.5
S4—Ni2—S5^ii^	101.04 (4)	S2—C4—H4C	109.5
S4^ii^—Ni2—S5^ii^	78.96 (4)	H4A—C4—H4C	109.5
S4—Ni2—S5	78.96 (4)	H4B—C4—H4C	109.5
S4^ii^—Ni2—S5	101.04 (4)	S3—C5—H5A	109.5
S5^ii^—Ni2—S5	180.0	S3—C5—H5B	109.5
S1—O1—Ni1	121.20 (14)	H5A—C5—H5B	109.5
S2—O2—Ni1	116.83 (15)	S3—C5—H5C	109.5
S3—O3—Ni1	122.93 (15)	H5A—C5—H5C	109.5
O1—S1—C2	104.45 (19)	H5B—C5—H5C	109.5
O1—S1—C1	105.4 (2)	S3—C6—H6A	109.5
C2—S1—C1	99.2 (3)	S3—C6—H6B	109.5
O2—S2—C3	104.06 (18)	H6A—C6—H6B	109.5
O2—S2—C4	104.50 (18)	S3—C6—H6C	109.5
C3—S2—C4	99.1 (2)	H6A—C6—H6C	109.5
O3—S3—C6	105.8 (2)	H6B—C6—H6C	109.5
O3—S3—C5	102.88 (19)	C8—C7—S5	125.7 (3)
C6—S3—C5	98.3 (2)	C8—C7—S4	125.1 (3)
C7—S4—Ni2	85.42 (14)	S5—C7—S4	109.2 (2)
C7—S5—Ni2	85.44 (14)	C7—C8—C9	121.2 (4)
S1—C1—H1A	109.5	C7—C8—C10	121.3 (4)
S1—C1—H1B	109.5	C9—C8—C10	117.5 (4)
H1A—C1—H1B	109.5	N1—C9—C8	179.0 (5)
S1—C1—H1C	109.5	N2—C10—C8	179.7 (6)
H1A—C1—H1C	109.5		
			
O3—Ni1—O1—S1	−142.43 (17)	Ni1—O3—S3—C5	−161.4 (2)
O3^i^—Ni1—O1—S1	37.57 (17)	S4^ii^—Ni2—S4—C7	−120 (100)
O2^i^—Ni1—O1—S1	127.71 (17)	S5^ii^—Ni2—S4—C7	173.58 (13)
O2—Ni1—O1—S1	−52.29 (17)	S5—Ni2—S4—C7	−6.42 (13)
O1^i^—Ni1—O1—S1	−139 (100)	S4—Ni2—S5—C7	6.44 (13)
O3—Ni1—O2—S2	−48.30 (17)	S4^ii^—Ni2—S5—C7	−173.56 (13)
O3^i^—Ni1—O2—S2	131.70 (17)	S5^ii^—Ni2—S5—C7	112 (14)
O2^i^—Ni1—O2—S2	13 (48)	Ni2—S5—C7—C8	170.5 (3)
O1—Ni1—O2—S2	−140.99 (17)	Ni2—S5—C7—S4	−8.58 (17)
O1^i^—Ni1—O2—S2	39.01 (17)	Ni2—S4—C7—C8	−170.5 (3)
O3^i^—Ni1—O3—S3	−178 (100)	Ni2—S4—C7—S5	8.59 (18)
O2^i^—Ni1—O3—S3	71.7 (2)	S5—C7—C8—C9	−177.2 (3)
O2—Ni1—O3—S3	−108.3 (2)	S4—C7—C8—C9	1.7 (6)
O1—Ni1—O3—S3	−18.3 (2)	S5—C7—C8—C10	1.6 (6)
O1^i^—Ni1—O3—S3	161.7 (2)	S4—C7—C8—C10	−179.4 (3)
Ni1—O1—S1—C2	145.1 (2)	C7—C8—C9—N1	26 (32)
Ni1—O1—S1—C1	−110.9 (3)	C10—C8—C9—N1	−153 (32)
Ni1—O2—S2—C3	149.93 (19)	C7—C8—C10—N2	79 (100)
Ni1—O2—S2—C4	−106.6 (2)	C9—C8—C10—N2	−102 (100)
Ni1—O3—S3—C6	95.9 (2)		

Symmetry codes: (i) −*x*+1, −*y*+1, −*z*+1; (ii) −*x*+2, −*y*, −*z*+1.
